# Anti-EBOV GP IgGs Lacking α1-3-Galactose and Neu5Gc Prolong Survival and Decrease Blood Viral Load in EBOV-Infected Guinea Pigs

**DOI:** 10.1371/journal.pone.0156775

**Published:** 2016-06-09

**Authors:** Olivier Reynard, Frédéric Jacquot, Gwénaëlle Evanno, Hoa Le Mai, Apolline Salama, Bernard Martinet, Odile Duvaux, Jean-Marie Bach, Sophie Conchon, Jean-Paul Judor, Andrea Perota, Irina Lagutina, Roberto Duchi, Giovanna Lazzari, Ludmilla Le Berre, Hélène Perreault, Elsa Lheriteau, Hervé Raoul, Viktor Volchkov, Cesare Galli, Jean-Paul Soulillou

**Affiliations:** 1 Molecular Basis of Viral Pathogenicity, CIRI, INSERM U1111—CNRS UMR5308, Université de Lyon, Université Claude Bernard Lyon 1, Ecole Normale supérieure de Lyon, Lyon, France; 2 Inserm-Jean Mérieux BSL4 Laboratory, US003 Inserm, Lyon, France; 3 Xenothera, Nantes, France; 4 INSERM, UMR 1064, Nantes, France; 5 CHU de Nantes, ITUN, Nantes, France; 6 Université de Nantes, Nantes, France; 7 IECM, EA4644 Université de Nantes, ONIRIS, USC1383 INRA, Nantes, France; 8 Avantea, Laboratory of Reproductive Technologies, Cremona, Italy; 9 Avantea Foundation, Cremona, Italy; 10 Chemistry Department, University of Manitoba, Winnipeg, Canada; 11 Department of Veterinary Medical Sciences, University of Bologna, Ozzano Emilia, Italy; Division of Clinical Research, UNITED STATES

## Abstract

Polyclonal xenogenic IgGs, although having been used in the prevention and cure of severe infectious diseases, are highly immunogenic, which may restrict their usage in new applications such as Ebola hemorrhagic fever. IgG glycans display powerful xenogeneic antigens in humans, for example α1–3 Galactose and the glycolyl form of neuraminic acid Neu5Gc, and IgGs deprived of these key sugar epitopes may represent an advantage for passive immunotherapy. In this paper, we explored whether low immunogenicity IgGs had a protective effect on a guinea pig model of Ebola virus (EBOV) infection. For this purpose, a double knock-out pig lacking α1–3 Galactose and Neu5Gc was immunized against virus-like particles displaying surface EBOV glycoprotein GP. Following purification from serum, hyper-immune polyclonal IgGs were obtained, exhibiting an anti-EBOV GP titer of 1:100,000 and a virus neutralizing titer of 1:100. Guinea pigs were injected intramuscularly with purified IgGs on day 0 and day 3 post-EBOV infection. Compared to control animals treated with IgGs from non-immunized double KO pigs, the anti-EBOV IgGs-treated animals exhibited a significantly prolonged survival and a decreased virus load in blood on day 3. The data obtained indicated that IgGs lacking α1–3 Galactose and Neu5Gc, two highly immunogenic epitopes in humans, have a protective effect upon EBOV infection.

## Introduction

The use of polyclonal antibodies has been the first breakthrough event in the treatment of life-threatening infectious diseases, including plague, diphtheria and cholera [1,2]. Despite the emergence of monoclonal antibodies, polyclonal antibodies from animal sources are still popularly used to treat toxin intoxication or as immunosuppressive agents in transplant recipients [3] or patients with autoimmune diseases [4]. Although animal-derived polyclonal antibodies have potential clinical advantages [5], an important limitation lies in their antigenicity, which results in the rapid, neutralizing immune response of the recipient towards the foreign IgG antigens. Indeed, all patients receiving animal polyclonal IgGs without other immunosuppression (IS) develop severe symptoms of immune-complex disease (serum sickness disease) [6]. The occurrence of these symptoms decreases with the strength of additional IS [6–8]. Thus, it is likely that the injection of high doses of animal IgGs will also result in severe serum sickness disease and the neutralization of their biological effects in the context of the prevention or treatment of severe infectious diseases. Furthermore, serum sickness disease symptoms, which include fever, arthralgia, pseudo-meningitis and skin eruptions, may mimic the symptoms of the severe infectious disease that is being prevented or cured.

The antigenicity of foreign IgGs arise from a combination of peptide and sugar antigens, which involve both the Fc and Fab parts of the IgGs in a polyclonal preparation [9,10]. In contrast, human antibodies do not express αGal nor Neu5Gc. Several attempts have aimed to reduce the immunogenicity of animal polyclonal IgGs, including the enzymatic removal of the Fc [11], the “humanization” of the Ig peptide backbone [12], or, as in this paper, the modification of the IgG glycans via knocking out the genes responsible for the expression of two key sugars that are recognized as major xeno-antigens by the human immune system (α1–3 Galactose, referred to as αGal [13], and the glycolyl form of neuraminic acid, referred to as Neu5Gc [14]).

EBOV belongs to the *Filoviridae* family, which comprises a group of enveloped negative-strand RNA viruses responsible for severe hemorrhagic fever in humans [15]. The EBOV genome is ~19 kb and encodes seven proteins that make up the virion: nucleoprotein (NP), virion proteins (VP) VP40, VP35, VP30, VP24, RNA-dependent RNA polymerase L and spike glycoprotein (GP). Surface GP is expressed as the result of transcriptional RNA editing [16] and is a highly N- and O-glycosylated type 1 glycoprotein composed of disulfide-linked subunits GP1 and GP2 generated by proteolytic cleavage of the GP precursor by the cellular protease furin [17]. EBOV GP is responsible for virus entry and is the target of virus-neutralizing antibodies [15]. Several publications have reported contrasting protective effects of convalescent serum [18–20] or monoclonal antibody cocktails [21] in curing or preventing EBOV infection, suggesting that animal-derived hyper-immune anti-EBOV polyclonal IgGs may also be useful [22]. By simultaneously targeting multiple epitopes, anti-EBOV polyclonal IgGs are also expected to prevent the generation of EBOV escape variants, a phenomenon already documented for this virus [23–25]. Several small animal models exist for EBOV infection, including mouse, guinea pigs and hamsters. Guinea pig infection with a well-characterized, adapted variant of EBOV induces a rapid and lethal disease state [26–28]. Therefore, this model has advantages compared to other rodent models and is useful for obtaining a proof of concept before the more ethically demanding primate model.

In this article, we aimed to provide a proof of concept that an anti-EBOV GP polyclonal IgG lacking αGal and Neu5Gc, and thus with a lower expected immunogenic potential in humans, can modify the course of an EBOV infection. Here, we show that double KO porcine IgGs lacking αGal and Neu5Gc prolong the survival of EBOV-infected guinea pigs and decrease EBOV replication in treated animals.

## Materials and Methods

### IB4 lectin and anti-Neu5Gc IgY binding on DKO IgGs

For the detection of αGal, ELISA plates were coated with DKO IgGs overnight at 4°C and were then blocked with PBS -Tween 0.1%- OVA 1% (Sigma-Aldrich, Saint Louis, MO, USA) for two hours at 37°C. After washing, the plates were incubated with Isolectin B4, peroxydase conjugated (IB4, 1/100 in PBSTO, Sigma Aldrich) for one hour at 37°C. After washing, the plates were developed using TMB substrate (Sigma-Aldrich), the reaction was stopped with H_2_SO_4_ 0.5 M, and the plate was read at 450 nm (reference filter 630 nm). For the detection of Neu5Gc epitopes, the plates were coated with DKO IgGs overnight at 4°C and blocked with PBS-Tween 0.1%-OVA 1%. After three washings, the plates were incubated with chicken IgY anti-Neu5Gc (1/1000 in PBSTO, Biolegend, San Diego, CA, USA) for one hour at room temperature. After washing, the plates were incubated with a goat anti-IgY-HRP (Abcam, Cambridge, UK) for one hour at room temperature. The results were developed as described above for anti-αGal.

### Mass spectrometry

The analysis of Neu5Gc, Neu5Ac, and αGal moieties on porcine IgGs was performed using mass spectrometry (MS), as previously described [29]. Briefly, double KO porcine IgGs (100μg) were reduced with dithiothreitol (Sigma-Aldrich), alkylated with iodoacetamide (Sigma-Aldrich), and digested with trypsin (4 μg, Promega, Madison, WI, USA). The digestion mixture was separated by reverse-phase HPLC. The pooled glycopeptide fractions were further digested with β-galactosidase from bovine testes (Prozyme, Hayward, CA, USA). All MS analyses were performed on an UltrafleXtreme mass spectrometer (Bruker Daltonics, Billerica, MA, USA) equipped with LID-LIFT^TM^ technology for tandem MS experiments. Dihydroxy-benzoic acid was used as the matrix.

### Ebola virus-like particles (VLPs) preparation

HEK293T cells were seeded in 150 cm² flasks and were transfected 24 hours later, at confluence ~80%, using Turbofect® (Thermo Fisher Scientific, Waltham, MA, USA) containing 20 μg phCMV-EBOV-VP40, 8 μg pCDNA3-EBOV-NP, 5 μg phCMV-EBOV-VP24 and 7 μg phCMV-EBOV-GP, according to the manufacturer’s recommendation. After 48 hours post-transfection, the supernatant was harvested, cleared from cell debris by low-speed centrifugation (5 minutes, 3000 g), and filtrated through a 0.45 μm filter. The particles were further pelleted at 250,000 g for 2 h in a SW32 rotor and Beckman LX100 ultracentrifuge and the pellet was then re-suspended in PBS. The total protein content was evaluated using a Protein Quantitation kit (Interchim, Montluçon, France), according to the manufacturer’s recommendation.

### DKO pig immunization (S1 Table)

A 14-month old male DKO pig weighing 102 kgs was obtained by cloning as described in [30], and was immunized with the VLPs at a dose of 700 μg in a 2 ml mix (v/v, 1:1) with Alhydrogel® adjuvant 2% (Invivogen, San Diego, CA, USA). Five intramuscular (IM) injections were performed in three different locations on days 0, 15, 29, 44 and 79. A 10 ml volume of blood was harvested on day 0, and after each immunization on days 15, 30, 57 and 83 to assess the antibody titers. On day 91, 100 ml of blood was taken for immunoglobulin extraction. All animal procedures were approved by the local Ethics Committee of the Laboratory of Reproductive Technologies, Avantea srl, and were carried out in accordance to the Italian regulation DGL 116/92.

### IgG purification

IgGs were purified on a Protein-A column (high performance Sepharose™, GE Healthcare, Little Chalfont, UK) using a low pressure chromatography and a 280 nm UV, pH and conductivity recorder. The immunoglobulins were eluted with a solution of 0.1 M citric acid pH 3, followed by an immediate pH neutralization of the eluate to pH 7–7.4 with a solution of 1 M TRIS pH 8. The IgGs were then dialyzed against PBS 1X and their amounts were assessed by spectrometry at 280 nm.

### Anti-EBOV IgG titers (ELISA) and neutralization assays

HEK 293T cells were transfected with phCMV GP. Transfected cells treated with 1% Triton were used as an antigen source for ELISA. Polysorp plates (Nunc, Thermo Fisher Scientific) were coated overnight at 4°C with the antigen (1:500 dilution), and incubated for 1 hour at 37°C in PBS containing 5% skimmed milk and 0.1% Tween 20 prior to incubation with dilutions of pig serum (in PBS containing 1% skimmed milk and 0.1% Tween 20). The presence of anti-EBOV antibodies was developed using an anti-pig HRP (Sigma-Aldrich) and TMB substrate (KPL, Gaithersburg, MD, USA).

The EBOV neutralization assay was based on the neutralization of a recombinant vesicular stomatitis virus (VSV) in which the gene coding for the VSV surface glycoprotein G was replaced with the EBOV GP gene. 200 plaque forming units (PFUs) of infectious VSV-EBOV-GP were incubated with consecutive two fold dilutions of the sera in 0.1 ml of DMEM for 30 min and were then used to inoculate Vero E6 cell cultures in eight replicates. After 1 hour, 5% FCS DMEM was added and the cells were incubated for 3 days prior to observation of the cytopathic effects by counter-staining with crystal violet. A 50% neutralizing titer was determined graphically as the sera dilution that neutralized the virus in 50% of the wells.

### Animal experiments: Assessment of DKO serum toxicity, guinea pigs groups and procedures (Table 1)

In order to test the safety of purified pig anti-EBOV IgGs obtained from double KO pigs (a pre-requisite from the ethical committee of the P4 facility to avoid unexpected toxicity of DKO IgGs for rodents), naïve male C57BL/6 mice of 8 weeks of age (Janvier Labs, Le Genest Saint Isle, France) were injected with the IgGs as following: two groups of 5 mice each received 2 mg of IgGs per day (corresponding to 400 mg/kg) intraperitoneally (IP) for 4 consecutive days. The mice were followed for changes in weight and general appearance for 1 month post-injection (S1 Fig).

**Table 1:** Design of the experimental procedures.

		Day 0	Day 0			Day 3		
Group number	Group name	EBOV injection	IgG injection	Route	Dose	IgG injection	Route	Dose
1	Mock-PBS	No injection	PBS	IM (in one leg)	n = 5 PBS	PBS	IM (in the other leg)	
2	EBOV + non-immune IgGs from DKO pig	1000 TCID50, IP	non-immune IgGs	IM (in the two legs)	n = 3, 65 mg, n = 1, 55.25 mg, n = 1, PBS	n.a.	n.a.	n.a.
3	EBOV + anti-Ebola IgGs from DKO pig (day 0)	1000 TCID50, IP	Anti-Ebola IgGs	IM (in one leg)	n = 5, 65 mg	n.a.	n.a.	n.a.
4	EBOV + anti-Ebola IgGs from DKO pig (day 0 and day 3)	1000 TCID50, IP	Anti-Ebola IgGs	IM (in one leg)	n = 5, 68 mg	Anti-Ebola IgGs	IM (in the other leg)	n = 4, 18.36 mg n = 1, 6.8 mg

Four groups of five guinea pigs were used as indicated. IP: intra-peritoneal, IM: intramuscular, n.a.: not applicable. Of note, the exact doses are indicated for each animal in each group. In group 2, one animal received a lower dose (55.25mg) than the first three (65mg), due to the limited amount of non-immune DKO IgGs available, the dead volumes of the syringe and the loss of material leading to a lower quantity of injected IgGs. One animal received PBS instead of the non-immune DKO pig IgGs. In group 4, the animals received lower doses on day 3 due to the limited amount of hyper-immune purified IgGs available: it was decided to give the highest dose at the first injection in order to have comparable doses in groups 3 and 4 at day 0.

The therapeutic efficacy of the anti-EBOV IgGs was tested in Hartley guinea pigs (150–200 g female guinea pigs, Harlan Laboratories, Netherlands) infected with recombinant guinea pig-adapted EBOV (EBOV 8MC passage 2) [26,27]. All experiments were carried out in the BSL-4 animal facility at the Inserm-Jean Mérieux BSL-4 laboratory in strict accordance with European directive 2010/63 and French regulations. The protocol was approved by the ethics committee for animal experimentation (Comité d’Evaluation Commun au Centre Léon Bérard, à l’Animalerie de transit de l’ENS, au PBES et au laboratoire P4 (CECCAPP) N°C2015; permit N°2015090209307871). Before handling, the animals were anesthetized in an induction box using isoflurane 3% under an air flow of 1 L/min. The animals were challenged through the IP route with 1000 TCID50 suspended in 0.3 ml DMEM. The animals were divided into 4 groups of 5 animals, and were treated as indicated in Table 1. The “Mock-PBS” group (Group 1) received 0.3 ml DMEM (IP) on day 0, and 1.5 ml PBS (IM in three different injection points) on days 0 and 3. The animals of the group 2 (“EBOV + non-immune IgGs from DKO pig”) were infected with EBOV on day 0. Three animals received 65mg of IgGs from a non-immunized DKO pig, one received 55.25mg, and the last animal received 1.5 ml of PBS. This difference was due to the limited amount of non-immune DKO IgGs available (n = 4 animals only on the group of 5 animals originally planned). In the group 3 (“EBOV + anti-Ebola IgGs from DKO pig (day 0)” group), all animals were infected at day 0 and received 65 mg of anti-EBOV IgGs the same day. All animals of the group 4 (“EBOV + anti-Ebola IgGs from DKO pig (day 0 and day 3)”) were infected on day 0; four animals were treated IM with 68 mg of anti-EBOV IgGs on day 0 and 18.36 mg of anti-EBOV IgGs on day 3, and one animal was treated with 68 mg on day 0 and 6.8 mg on day 3. The differences between the doses at day 0 and day 3 were due to the limited total amount of hyper-immune purified IgGs: the highest dose was given at the first injection on day 0, in order to have comparable doses in groups 3 and 4 on day 0. The animals were monitored for symptoms, weight and temperature every day from day 3 post-infection until death or euthanasia at the end point corresponding to a 20% weight loss. Animal sacrifice was performed under isoflurane anesthesia by intra-cardiac injection of pentobarbital.

Blood samples were obtained on days 0 and 3 from all animals for viral load analysis. The possible toxicity of the anti-EBOV IgGs, as a potential confounding factor of DKO IgG activity, was ruled out in C57/B6 mice injected IP with a dose of 400 mg/kg.

### Viral RNA level assessment

Viral RNA was extracted from the sera of EBOV-infected guinea pigs (Qiagen viral RNA extraction kit, Hilden, Germany) and analyzed using a one-step SYBR green RTqPCR kit (Eurobio, Les Ulis, France) and primer pairs: EBOV Forward 5’CGGAGGCTTTAACCCAAATA (L polymerase, position 14870) and EBOV Reverse 5’TCATACATGGGAGTGTGGCT (L polymerase, position 14987). The analyses were performed on a Roche LC96 real time PCR apparatus. Quantification was performed using a range of dilutions of EBOV DNA and expressed as relative copy number per ml (detection limit of the technique is 180 relative copies per ml).

### Statistical analysis

Survival in the different groups was compared using Kaplan-Meier analysis and was analyzed using a log rank test. Weight loss curves were analyzed using a repeated measures two-way ANOVA test. In the group with two anti-EBOV IgG injections, the virus loads on day 3 (before the second injection of IgGs) were compared using a non-parametric Mann Whitney test. The correlation between virus load and survival time was analyzed using Kendall's rank correlation coefficient.

## Results

### Absence of αGal and Neu5Gc on DKO IgG

Two ELISAs were used for the detection of Neu5Gc and αGal expression on double KO pig IgGs, in which the IgGs were immobilized on the plates and detected using either chicken anti-Neu5Gc IgY antibodies or αGal-binding isolectin B4. No Neu5Gc or αGal expression was detected on the double KO pig IgGs (data not shown). In addition, as seen in S2 Fig, the MS analysis confirmed that the IgGs from GT1 and CMAH double KO pigs lacked the αGal and Neu5Gc epitopes. Double KO porcine IgGs only displayed the Neu5Ac form of neuraminic acid. Moreover, there was no detectable Galα-1,3Gal-β-1,4GlcNAc branching, as all Gal residues were hydrolyzed by -galactosidase, confirming that the DKO IgGs did not express the two xeno-epitopes.

### Anti-EBOV titers and virus neutralization test

**Fig 1:**
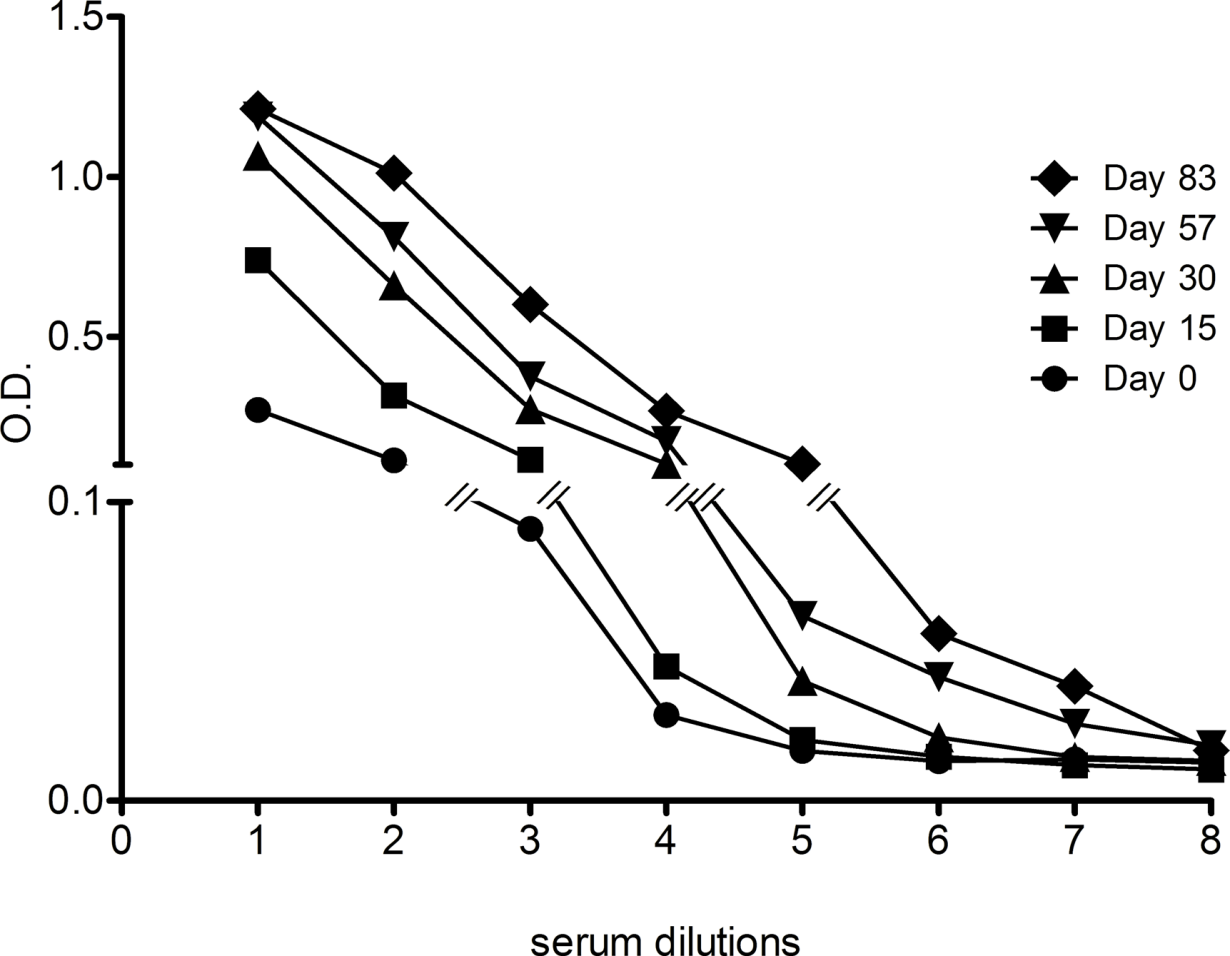
Effect of the immunization of double KO pig with EBOV VLPs. Serum samples obtained from an immunized double KO pig before (day 0), during (day 15, 30 and 57) or after immunizations (day 83) were analyzed using ELISA for specific anti-GP antibodies. The mean OD at each time point for each dilution is represented. Anti-EBOV GP titers of the latest collected pig sera was about 1:100,000.

Over an 83 day-period, a single double KO pig was immunized five times with 700 μg EBOV VLPs generated upon the co-expression of the viral proteins VP40, VP24, and NP and the surface glycoprotein GP. An analysis of immunization profile showed that each injection was beneficial in terms of an increase in anti-EBOV antibody titers, with a maximum ELISA titer of 1:100,000 on day 83 (Fig 1). The titer was determined as the last dilution with a signal over the value of the mock control plus two times the standard deviation. Anti-EBOV neutralization tests were performed with the sera collected on days 57 and 83 using an SN50 assay with a recombinant VSV carrying EBOV GP (see the Materials and Methods section). Neutralization titers of the sera were 1:40 and 1:100, respectively.

### Protective effect of anti-EBOV IgGs

**Fig 2:**
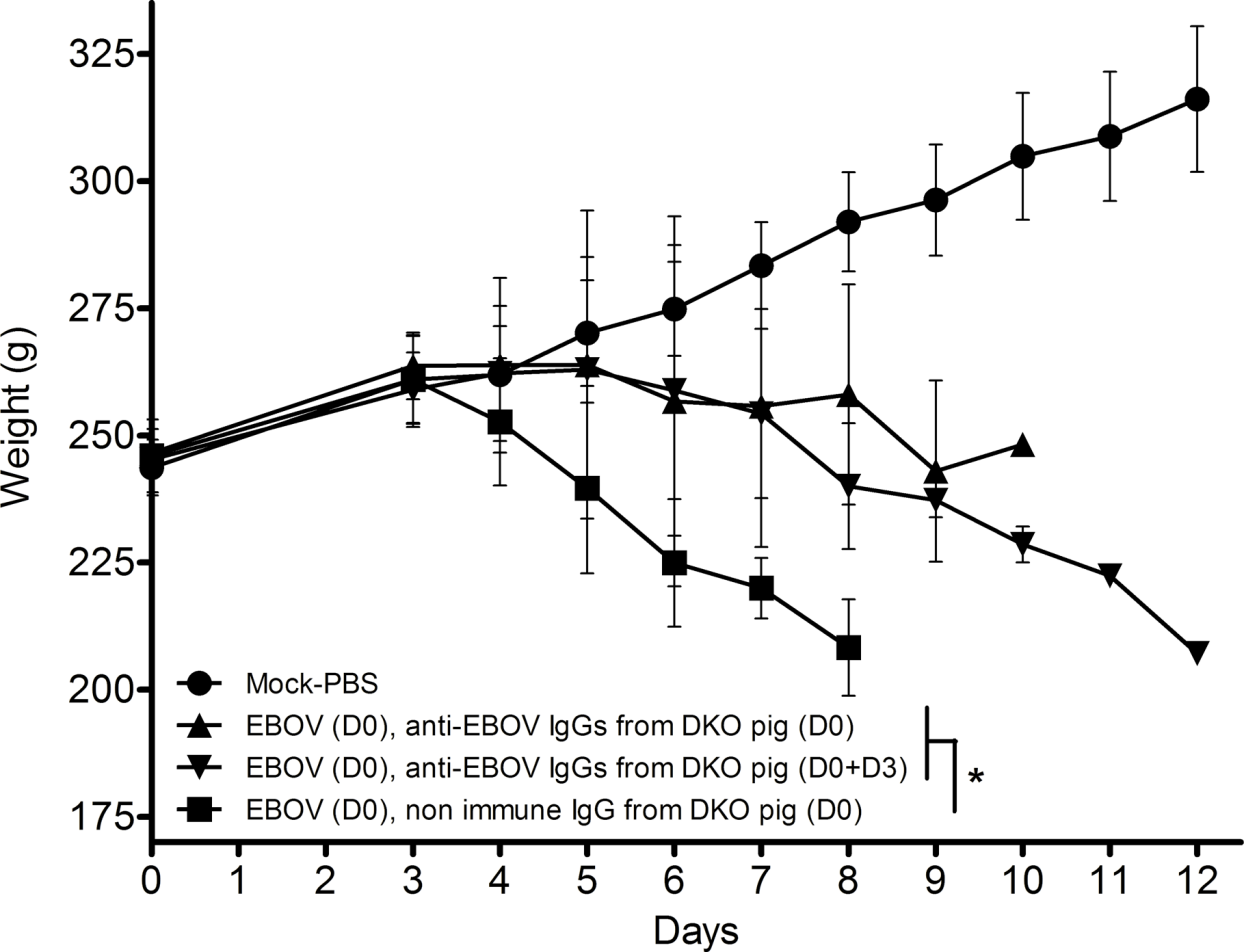
Monitoring of the weight of guinea pigs treated with anti-EBOV IgGs. Each curve represents one group of 5 guinea pigs. The standard deviation is represented for each group at each time point. * p<0.05 when comparing all guinea pigs having received anti-EBOV IgGs to animals having received IgGs from a non-immunized pig, using a repeated measures two-way ANOVA test.

**Fig 3:**
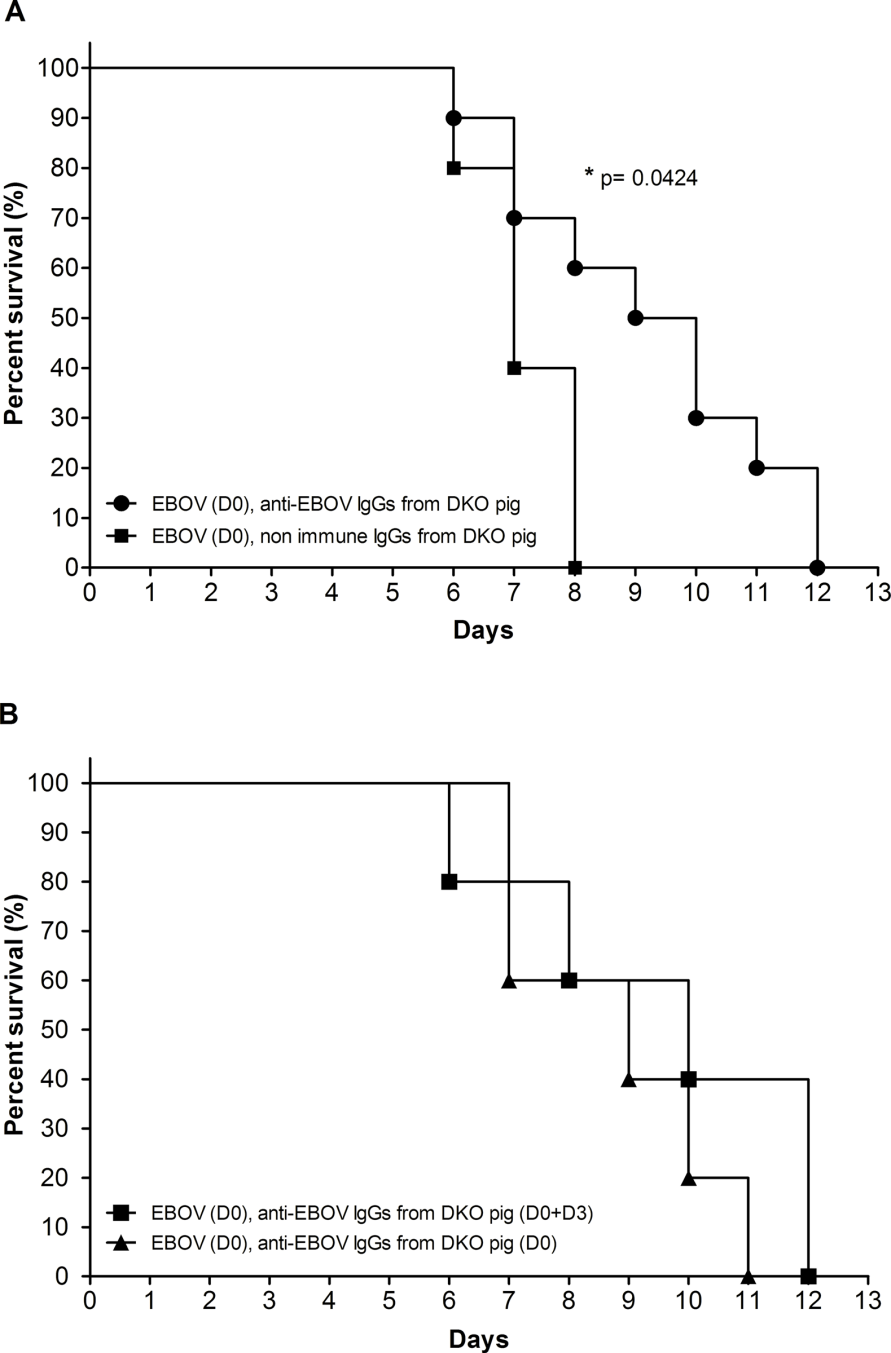
Kaplan Meier survival curves of guinea pigs after EBOV infection, according to treatment with anti-EBOV IgGs. A: Survival of guinea pigs having received the anti-EBOV IgGs (n = 10) compared to guinea pigs receiving the non-immune DKO IgGs (n = 5, *p = 0.0424, using a Log Rank test). B: Survival of guinea pigs having received one (DO) or two (D0 and D3) doses of polyclonal anti-EBOV IgGs.

The design of the experimental procedure is given in Table 1. Fig 2 shows that the weights of the guinea pigs that received the injections of anti-EBOV IgGs on day 0 only or on days 0 and 3 were significantly different (p<0.05) from the weights of the animals that received IgGs from the non-immunized double KO pig, suggesting that the course of the disease was less vigorous in the treated animals. However, the weight curves were similar in the groups that received one dose on day 0 or two doses on days 0 and 3. Fig 3 shows the Kaplan-Meier survival curve of the animals in the various experimental groups. Three animals of group 3 were found dead on days 7, 10 and 11 post-injection, and one from group 4 was found dead on day 7. These animals displayed Ebola virus disease symptoms, notably a high fever, the day before death but had not yet reached the end point criteria at that time. No samples were collected from dead bodies. There was a significant difference (p = 0.042, log rank test) in the survival time of the animals that received the anti-EBOV IgGs compared to the untreated group. However, no further improvement was observed in the animal receiving the two anti-EBOV IgG injections on days 0 and 3. No animal survived the EBOV infection after day 12.

In order to evaluate the toxicity of the hyper-immune IgGs, mice were challenged with a high dose of DKO IgGs (400 mg/kg). All animals had a stable weight (S1 Fig) and normal activities without clinical signs of toxicity up until the end of the follow-up at one month post-injection, when the animals were euthanized. These data indicate that the deaths in guinea pigs that received the virus and the anti-EBOV IgGs were not due to IgG toxicity.

### Blood viral genome load

**Fig 4:**
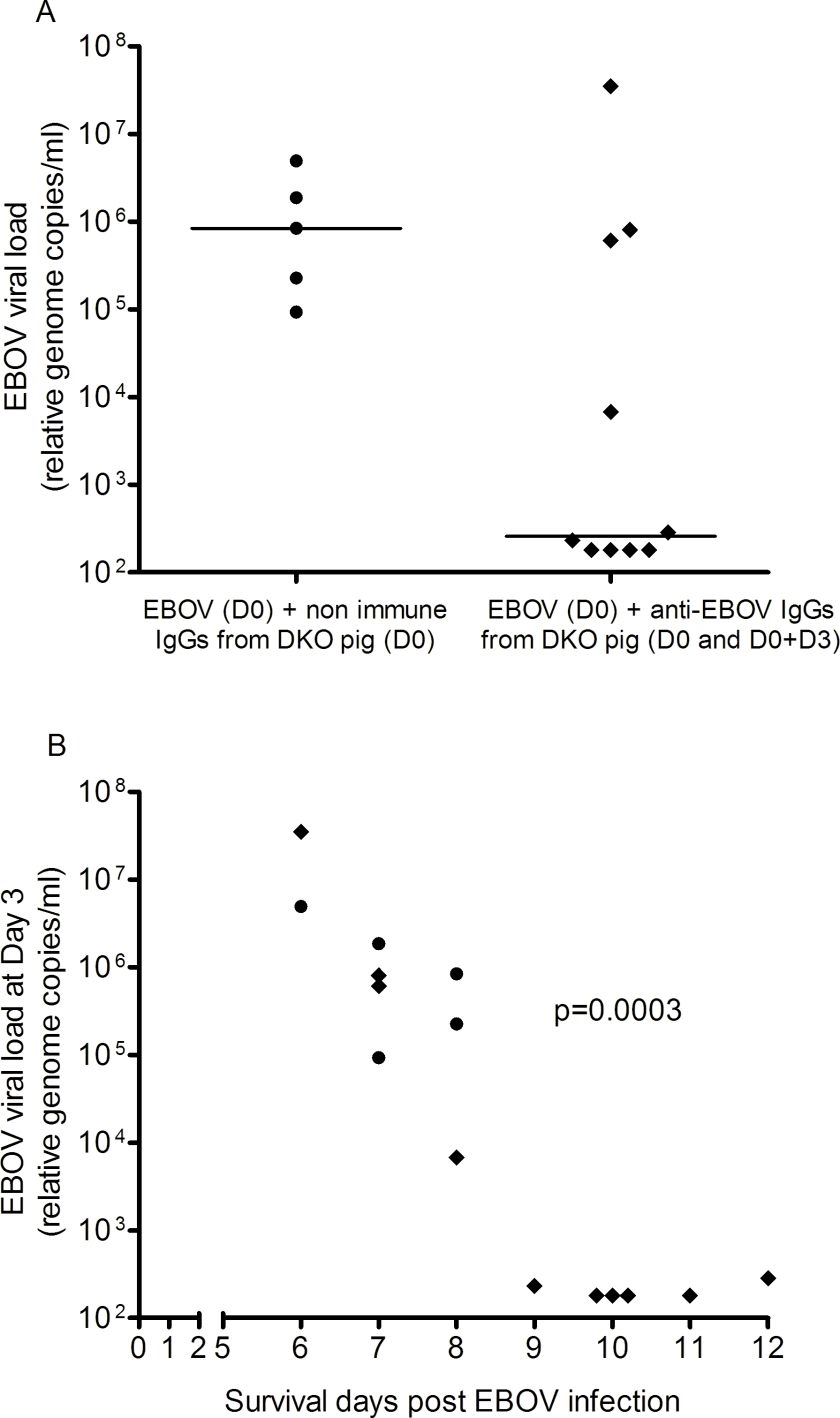
Viral loads in serum at day 3 post-EBOV infection. A: Levels of circulating EBOV transcripts at day 3 post infection in the serum of each animal was evaluated by RT-qPCR using primers targeting EBOV polymerase gene. The horizontal bars represent the median values. The median D3 virus load was 839,333 following the injection of non-immune IgGs from a DKO pig and 259 following the injection of anti-EBOV antibodies (p = 0.055, using a Mann-Whitney test). The limit of detection of the test was of 180 relative genome copies/ml, and all values under this threshold were considered as negative data (as represented on the graph). B: Correlation between viral load on day 3 and guinea pig survival. Kendall's rank correlation coefficient showed a significant negative correlation (p = 0.0003) between EBOV viral load at day 3 and survival following infection, when considering all pooled data. Non-treated animal’s values are displayed as circles whereas treated animal’s values are displayed as squares.

Fig 4A shows the level of EBOV transcripts in circulating blood in the various groups of animals. Values under the detection limit of the test (180 relative genome copies/ml) were considered as negative values. There was a low but borderline significance (Mann Whitney test, p = 0.055) of the viral genome load in the anti-EBOV IgG treated guinea pigs compared to the untreated animals, with a 3-log median value decrease obtained on day 3 post-infection (i.e., as assessed in 10 animals, in the serum harvested before the second injection of anti-EBOV IgGs in group 4). Furthermore, as shown in Fig 4B, there was a significant correlation between the level of virus load and the survival time of the treated animals (Kendall's rank correlation coefficient, p = 0.0003). Altogether, the data show that the anti-EBOV IgGs from the DKO pigs could delay the death of the treated guinea pigs and significantly decrease the circulating virus loads at an early time point following infection.

## Discussion

Polyclonal IgGs offer several theoretical advantages compared to monoclonal antibodies by displaying an extended repertoire and functional capacity [1,5,31]. Due to the high diversity of the epitopes recognized by polyclonal IgGs and the possibility to build platforms of animal immunization against viral variants, polyclonal IgGs may also alleviate the problem in which viral mutants can escape the effects of individual antibodies. Data on the efficacy of polyclonal preparations to prevent or cure Ebola disease in humans or animal models are conflicting. The treatment of laboratory-acquired infection using convalescent phase serum [18] and early reports of a survival of 7 out of 8 patients after blood transfusions from convalescent patients [19] spurred further experimental studies, although this last study did not reach statistical significance, and was limited by a late treatment of the patients following exposure to the virus [32]. Nevertheless, blood transfusion from convalescent-phase monkeys immunized against EBOV failed to prolong the survival of infected animals [33]. However, hyper-immune horse sera with in vitro neutralizing capacities have shown its capability of reducing viremia in an experimental model of the disease [34], and in some studies, purified polyclonal antibodies from convalescent primates totally protected infested animals following infusion performed immediately or 48 hours after infection [35]. Furthermore, ovine anti-EBOV hyper-immune IgGs with a high anti-EBOV neutralization titer, induced full protection against EBOV in guinea-pigs [22]. Although this study differs from ours, with a possibly less virulent EBOV infection model (as suggested by an average survival time of infected guinea pigs of 11 days versus 9 days using another guinea pigs adapted virus [36]), these data strongly suggest that a passive protection strategy may be successful. Our study also differs by a different virus (IP) and antibody (IM) injection route, aiming to decrease immediate interference between the two moieties which may artificially improve the efficiency of the drug, although an IV route might have been more pertinent when considering the systemic disease model used here. However, this approach using unmodified ovine IgGs is also likely to generate a strong immune response against αGal and Neu5Gc epitopes. Other attempts to passively protect animals from an experimental disease using polyclonal antibodies from convalescent animals have also yielded conflicting results [23,37,38]. Recent data regarding the use of passive plasma-therapy in humans have not been definitive [20].

In this article, we aimed to study the efficacy of polyclonal IgGs obtained from genetically modified pigs, and directed against VLPs displaying several EBOV proteins, on the survival of guinea pigs infected with EBOV. These “humanized” polyclonal IgGs lack αGal and Neu5Gc, two major xenoantigens for the human immune system. These two sugars are not expressed specifically in human beings (and some new world monkeys), due to a loss mutation, however they are naturally expressed in guinea pigs [39]. Therefore, using such IgGs in a guinea pig model does not impact their potential immunogenicity, and our model of infection does not highlight at full degree the importance of “glycan-humanized” porcine IgGs for disease treatment. Rather, we aimed to explore here whether the modifications in these IgG molecules did not alter their in vivo efficacy in an experimental EBOV disease.

In the context of EBOV disease development in humans, these modified IgGs would not be recognized by preexisting anti-αGal [13] (including of IgE isotype [40]) or anti-Neu5Gc antibodies, which are present in most normal individuals as a result of immunization by the gut flora (for αGal) [13,41], or by diet (for Neu5Gc) [42,43].

As the infusion of unmodified animal polyclonal IgGs in almost all cases results in serum sickness disease [6], there have been several attempts to decrease the expected immunogenicity of animal hyperimmune IgGs against life-threatening viruses, including EBOV. For instances, partially “humanized” bovine IgG protein backbones have been obtained from genetically engineered animals [12,44]. Moreover, truncated IgG fragments lacking Fc [11] have been proposed. However, these “humanized” bovine IgGs still display substantial stretches of bovine peptide sequences that do express Neu5Gc and αGal. Truncated polyclonal animal preparations lack the major functions of IgGs related to complement activation and Fc-gIII-R binding, which are likely important in the protection against the virus. In addition, the F(ab)’2 preparation is not deprived of the glycans displayed by hyper-variable regions in approximately 20–40% of the polyclonal molecules [9,10].

The DKO pig mounted a vigorous humoral immune response against the VLPs, although the antigen preparation may not be optimal compared to, for instance, VSV-EBOV GP (an antigen which was not used due to its possible pathological hazards in pigs [45]). The “orphan” situation of SIGLECs, the natural ligands of Neu5Gc [39], results from the CMAH KO and may be favorable for improving the pig immune response against viral antigens since the SIGLEC/Neu5Gc interaction is a strong inhibitor of B cell activation. The neutralization titers of our anti-Ebola serum remained modest, although the different methods of quantification used for the measurement of antibody titers are difficult to compare. Refinements of the immunization procedure and the antigen preparation may improve efficiency and allow better results. However, the significant effect of the DKO IgGs on the survival and virus load in the guinea pig, despite a low titer of neutralizing antibodies, suggests the possible effect of an increased complement binding compared to wild-type pig IgGs ([46], Salama A et al., manuscript in preparation). This is also consistent with the fact that the neutralization tested on the pig serum was found to be complement-dependent, and disappeared after heat inactivation.

In this study, an in vivo assessment of the anti-EBOV IgGs therapeutic effect was performed using a guinea pig model [26,47] and a well characterized guinea pig-adapted EBOV [27]. This model allowed for the IM injection of the anti-EBOV IgGs in a different site from that of the virus injection. This is in contrast to reports in the mouse model, in which both the EBOV and the anti-EBOV preparation were injected IP [48]. First, we showed that fatality and weight loss in the infected guinea pigs injected with the genetically modified anti-EBOV IgGs did not result from a possible toxicity of the injected IgG preparation. In a separate experiment using mice we demonstrated that 400mg/kg of anti-EBOV IgGs neither affected the survival nor the weight of the animals. Moreover we showed that the injection of anti-VLP IgGs could significantly prolong the survival times of infected guinea pigs, although no animal ultimately survived the virus injection. A second injection of anti-EBOV IgGs did not result in any further significant prolongation of survival, which could be partly explained by the lower dose of IgGs given at day 3.

Viremia in patients with EBOV diseases is considered to be a strong predictor of death [49–51]. The blood virus load measurements showed both a 3-log median value decrease in virus transcript levels (Fig 4A) on day 3 (i.e., before the second IgG injection; as assessed in 10 animals) but also a significant difference between the animals in the circulating EBOV. Apparently the 3-log drop in circulating EBOV was not sufficient to allow survival. Most likely continuous virus replication and, importantly, the release into the blood of soluble viral glycoproteins capable of neutralizing at least some of anti-EBOV IgGs [52,53] could explain why this current design to cure an infection showed only a modest success. However strong viral load diminution induced by the polyclonal IgGs suggests that this treatment may offer an early anti-viral synergy when associated with higher dose of anti-EBOV IgGs, replication inhibitors or a vaccine.

Although our conclusions need to be confirmed using a greater number of animals and increased neutralizing titers, our experiments altogether suggest that IgGs lacking Neu5Gc and αGal can modify the survival and virus load following a lethal injection of EBOV in guinea pigs. These data encourage us to use these genetically modified animals to prepare hyper-immune anti-EBOV IgGs of higher virus neutralizing titers for future studies in guinea pig and cynomolgus models before considering a clinical application.

## Supporting Information

S1 Fig Follow-up weights of mice receiving a high-dose of anti-EBOV IgGs. 8-week old C57BL/6 mice were treated IP with 2 mg of IgGs per day (400 mg/kg) for four consecutive days.
(DOCX)

S2 Fig MALDI-TOF mass spectrometry of glycopeptides from double knock-out IgGs. A: negative ionization, linear detection mode highlighting the presence of N-acetyl neuraminic acid and the absence of N-glycolylneuraminic acid; B: positive ionization, reflector detection mode prior to treatment of the sample with β-galactosidase; C: positive ionization, reflector detection mode after β-galactosidase treatment. The effect of β-galactosidase on the different glycoforms is shown by the arrows. All terminal galactosyl residues are removed by the enzyme, indicating the absence of galactosyl residues linked in α1,3.
(DOCX)

S1 Table Double KO pig immunization protocol. A DKO pig was immunized with five doses of 700μg VLPs each. Injections were done intramuscularly on days 0, 15, 29, 44 and 79. A 10 ml volume of blood was harvested on day 0 and on days 15, 30, 57 and 83 after each immunization assess the anti-EBOV antibody titers. On day 91, 100 ml of blood were taken for IgG extraction.
(DOCX)

## Abbreviations

αGal: Galactose-α1–3 Galactose
EBOV: Ebola virus
GP: glycoprotein
IM: intra-muscular
IP: intra-peritoneal
IS: immunosuppression
KO: knock-out
MS: mass spectrometry
Neu5Gc: glycolyl form of the neuraminic acid
NP: nucleoprotein
PFU: plaque forming unit
VLP: virus-like particle
VP: virion protein
VSV: vesicular stomatitis virus
