# Supplementary material for: Anti-EBOV GP IgGs Lacking α1-3-Galactose and Neu5Gc Prolong Survival and Decrease Blood Viral Load in EBOV-Infected Guinea Pigs
Source: PLoS One. 2016 Jun 9;11(6):e0156775. doi: 10.1371/journal.pone.0156775 (PMC4900587; doi:10.1371/journal.pone.0156775)
Supplement: S1 Fig — 8-week old C57BL/6 mice were treated IP with 2 mg of IgGs per day (400 mg/kg) for four consecutive days. (DOCX) [file pone.0156775.s001.docx]

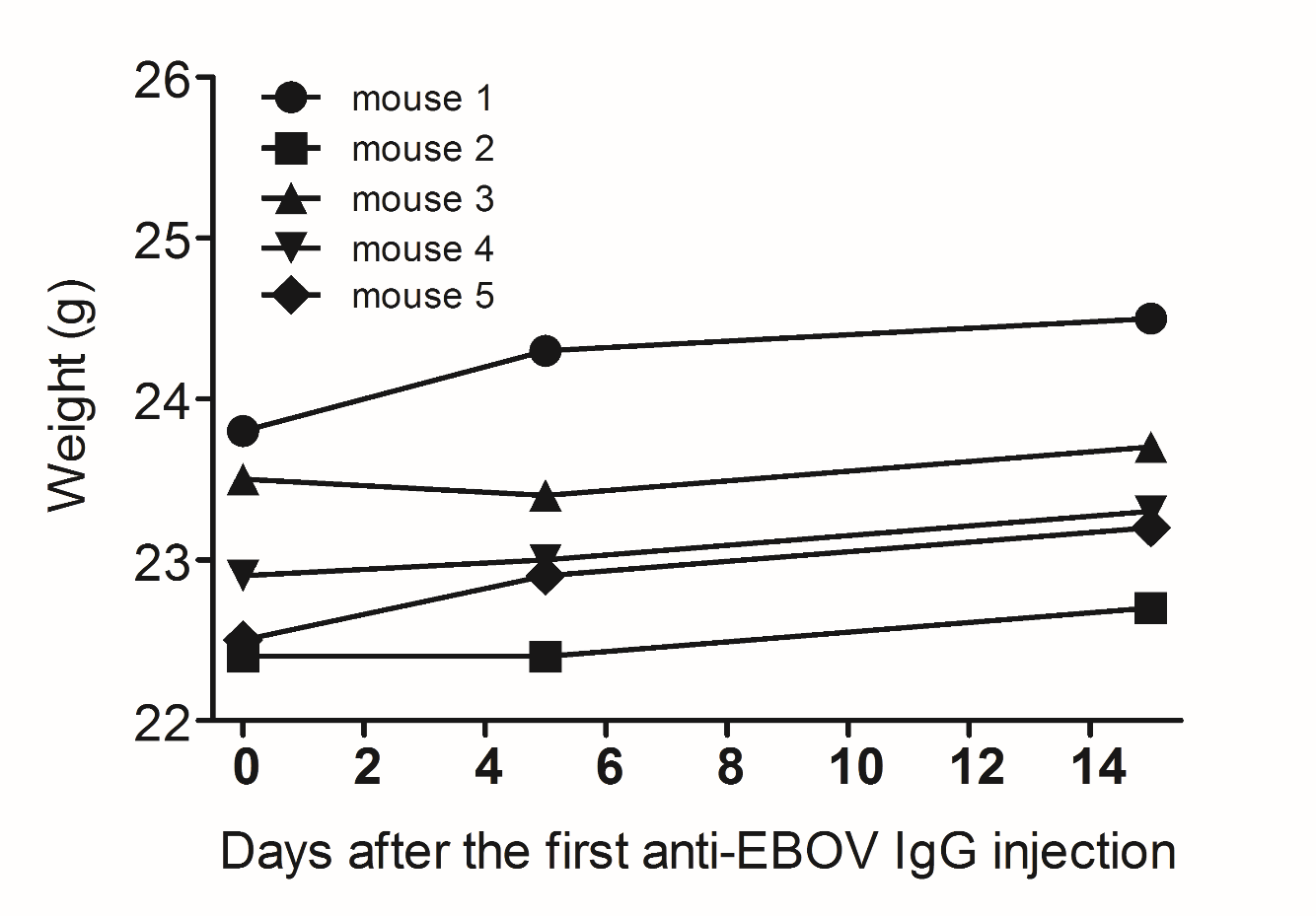


**S1 Fig. Follow-up weights of mice receiving a high-dose of anti-EBOV IgGs.** 8-week old C57BL/6 mice were treated IP with 2 mg of IgGs per day (400 mg/kg) for four consecutive days.
