# Supplementary material for: Anti-EBOV GP IgGs Lacking α1-3-Galactose and Neu5Gc Prolong Survival and Decrease Blood Viral Load in EBOV-Infected Guinea Pigs
Source: PLoS One. 2016 Jun 9;11(6):e0156775. doi: 10.1371/journal.pone.0156775 (PMC4900587; doi:10.1371/journal.pone.0156775)
Supplement: S2 Fig — A: negative ionization, linear detection mode highlighting the presence of N-acetyl neuraminic acid and the absence of N-glycolylneuraminic acid; B: positive ionization, reflector detection mode prior to treatment of the sample with β-galactosidase; C: positive ionization, reflector detection mode after β-galactosidase treatment. The effect of β-galactosidase on the different glycoforms is shown by the arrows. All terminal galactosyl residues are removed by the enzyme, indicating the absence of galactosyl residues linked in α1,3. (DOCX) [file pone.0156775.s002.docx]

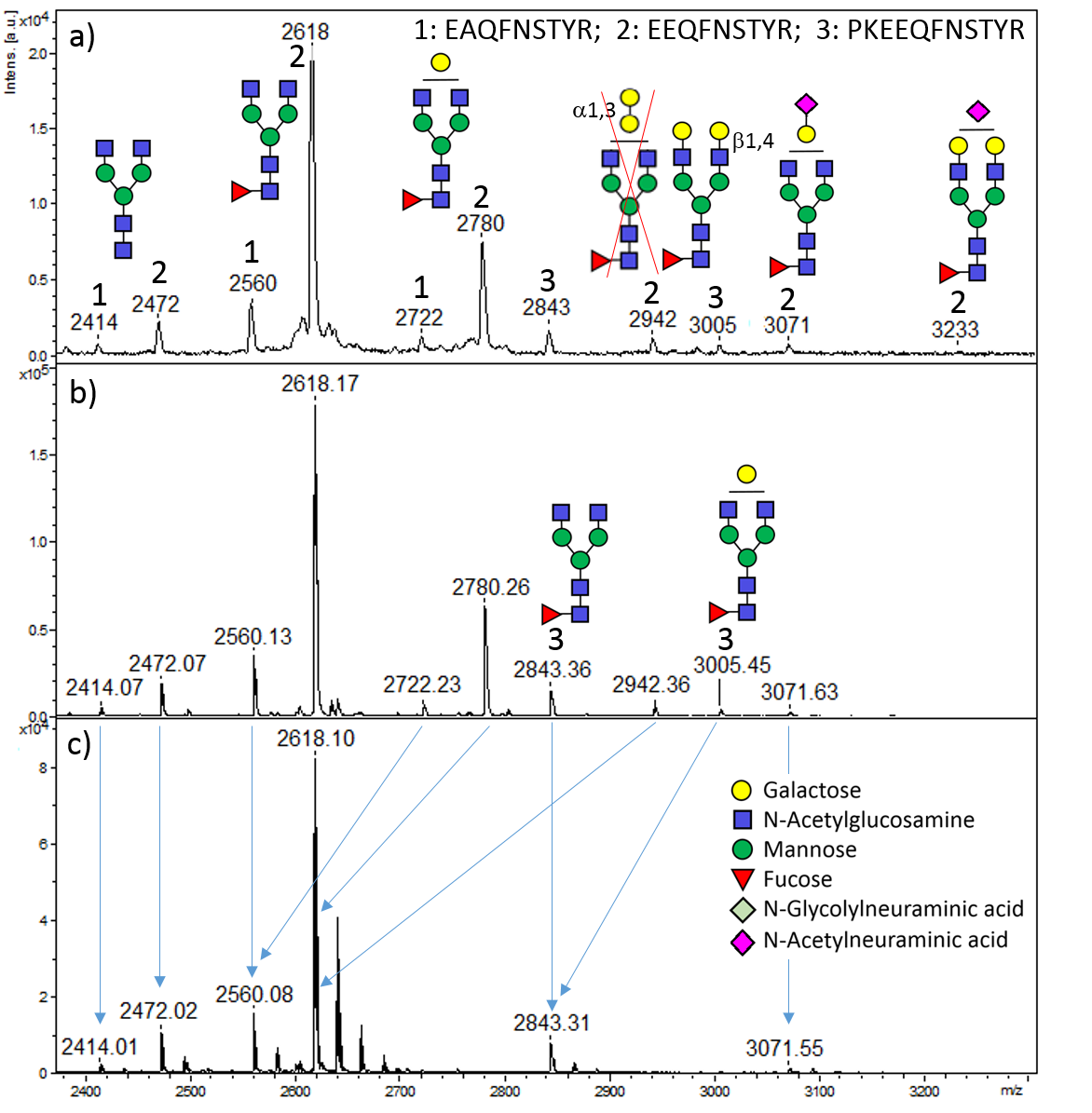


**S2 Fig. MALDI-TOF mass spectrometry of glycopeptides from double knock-out IgGs.** A: negative ionization, linear detection mode highlighting the presence of N-acetyl neuraminic acid and the absence of N-glycolylneuraminic acid; B: positive ionization, reflector detection mode prior to treatment of the sample with β-galactosidase; C: positive ionization, reflector detection mode after β-galactosidase treatment. The effect of β-galactosidase on the different glycoforms is shown by the arrows. All terminal galactosyl residues are removed by the enzyme, indicating the absence of galactosyl residues linked in α1,3.
