# Supplementary material for: Anti-EBOV GP IgGs Lacking α1-3-Galactose and Neu5Gc Prolong Survival and Decrease Blood Viral Load in EBOV-Infected Guinea Pigs
Source: PLoS One. 2016 Jun 9;11(6):e0156775. doi: 10.1371/journal.pone.0156775 (PMC4900587; doi:10.1371/journal.pone.0156775)
Supplement: S1 Table — A DKO pig was immunized with five doses of 700μg VLPs each. Injections were done intramuscularly on days 0, 15, 29, 44 and 79. A 10 ml volume of blood was harvested on day 0 and on days 15, 30, 57 and 83 after each immunization assess the anti-EBOV antibody titers. On day 91, 100 ml of blood were taken for IgG extraction. (DOCX) [file pone.0156775.s003.docx]

**S1 Table. Double KO pig immunization protocol.** A DKO pig was immunized with five doses of 700 μg VLPs each. Injections were done intramuscularly on days 0, 15, 29, 44 and 79. A 10 ml volume of blood was harvested at day 0, and after each immunization on days 15, 30, 57 and 83 to assess the anti-EBOV antibody titers. On day 91, 100 ml of blood were taken for IgG extraction.

| **Intraperitoneal Ebola-VLP injections (700 µg)** | **Blood samplings (10 ml) to assess anti-EBOV IgG titers** | **Blood harvesting (100 ml) to extract IgGs** |
| --- | --- | --- |
|  |  |  |
| Day 0 | Day 0 | Day 91 |
| Day 15 | Day 15 |  |
| Day 29 | Day 30 |  |
| Day 44 | Day 57 |  |
| Day 79 | Day 83 |  |
